# Properties of Biodegradable Films Based on Poly(butylene Succinate) (PBS) and Poly(butylene Adipate-*co*-Terephthalate) (PBAT) Blends ^†^

**DOI:** 10.3390/polym12102317

**Published:** 2020-10-10

**Authors:** Anna Raffaela de Matos Costa, Andrea Crocitti, Laura Hecker de Carvalho, Sabrina Carola Carroccio, Pierfrancesco Cerruti, Gabriella Santagata

**Affiliations:** 1 Department of Chemical Engineering, Federal University of Pernambuco, Recife 50670-901, Brazil; raffaela_matos@yahoo.com.br; 2 Department of Industrial Engineering, University of Salerno, 84084 Fisciano, Italy; andrea.crocitti@gmail.com; 3 Departament of Materials Engineering, Federal University of Campina Grande, Campina Grande 58429-900, Brazil; 4 Institute for Polymers, Composites and Biomaterials (IPCB-CNR), 9-95126 Catania, Italy; sabrinacarola.carroccio@cnr.it; 5 Institute for Polymers, Composites and Biomaterials (IPCB-CNR), I-80078 Pozzuoli, Italy; gabriella.santagata@ipcb.cnr.it

**Keywords:** biodegradable polymer blends, poly(butylene succinate) (PBS), poly(butylene adipate-*co*-terephthalate) (PBAT), mechanical properties, rheological properties, water vapor and oxygen permeability

## Abstract

Compression molded biodegradable films based on poly(butylene succinate) (PBS) and poly(butylene adipate-*co*-terephthalate) (PBAT) at varying weights were prepared, and their relevant properties for packaging applications are here reported. The melt rheology of the blends was first studied, and the binary PBS/PBAT blends exhibited marked shear thinning and complex thermoreological behavior, due to the formation of a co-continuous morphology in the 50 wt% blend. The films were characterized by infrared spectroscopy (FTIR), differential scanning calorimetry (DSC), mechanical tensile tests, scanning electron microscopy (SEM), and oxygen and water vapor permeability. PBS crystallization was inhibited in the blends with higher contents of PBAT, and FTIR and SEM analysis suggested that limited interactions occur between the two polymer phases. The films showed increasing stiffness as the PBS percentage increased; further, a sharp decrease in elongation at break was noticed for the films containing percentages of PBS greater than 25 wt%. Gas permeability decreased with increasing PBS content, indicating that the barrier properties of PBS can be tuned by blending with PBAT. The results obtained point out that the blend containing 25 wt% PBS is a good compromise between elastic modulus (135 MPa) and deformation at break (390%) values. Overall, PBS/PBAT blends represent an alternative for packaging films, as they combine biodegradability, good barrier properties and reasonable mechanical behavior.

## 1. Introduction

The quest for the development of biobased and biodegradable polymer formulations is ever increasing due to environmental pollution, global heating and the foreseen shortage of oil supplies. In this respect, biodegradable and partially biobased polyesters, such as poly(lactic acid) (PLA), poly(butylene succinate) (PBS) and aliphatic-aromatic copolyesters, are considered as suitable replacements for traditional oil-based commodity polymers, such as polyolefins or poly(ethylene terephthalate) (PET) [1,2,3,4,5]. Compared to the latter, the main drawbacks of biobased and biodegradable polymers concern the requirements of strictly controlled processing conditions and increased thermal sensitivity, poor barrier and mechanical properties, and feedstock availability being subject to seasonal variation. Therefore, many research efforts focus on the development of biodegradable polymer blend formulations with tailored thermal, rheological and mechanical behavior.

**Figure 1:**
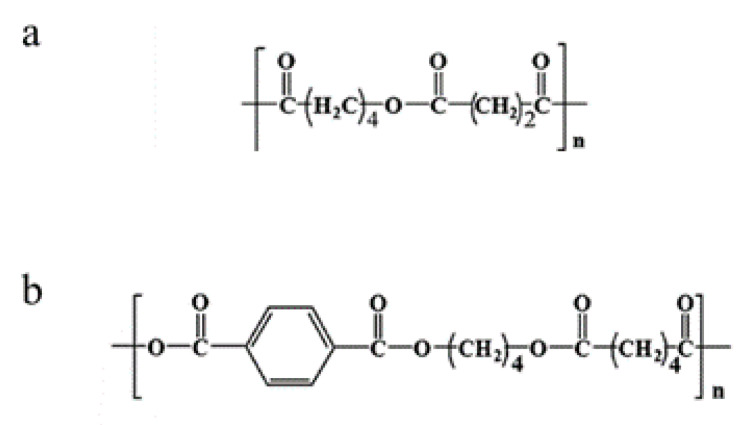
Chemical structure of (**a**) PBS and (**b**) PBAT.

PBS (Figure 1a) is an aliphatic polyester synthesized by polycondensation between succinic acid and 1,4-butanediol. It can be produced from renewable resource-based succinic acid obtained by the bacterial fermentation of sugars, glucose, starch, xylose, etc. [1]. Due to its semi-crystalline nature, thermal stability, melting point lower than that of other commercially available biodegradable polymers, and easy processing with conventional film casting and blowing techniques, PBS is an excellent candidate for the production of biodegradable films for packaging. In particular, PBS films have good barrier properties, as PBS’s oxygen permeability (*P*_O_) is about four times lower than that of low-density polyethylene (LDPE) and about double that of PLA [2,5]. Furthermore, PBS’s mechanical properties are similar to those of conventional polyolefins, with good tensile strength and moderate stiffness and hardness. In comparison to PLA, PBS can be characterized as a tough and resistant material [6]. However, its ductility is rather poor, and therefore several studies on PBS blends with other polymers have been carried out in order to modify its properties [6,7,8,9,10,11]. In this respect, blending with other biodegradable polyesters is considered as the most promising way to get sustainable materials with improved and tailored properties. John et al. [12] noticed a considerable improvement in the mechanical properties of PBS/PCL blends due to increased crystallinity, related to the interaction of the polymers’ ester groups through hydrogen bonds. Qiu et al. [13] investigated the properties of the PLA/PBS blends and concluded that PBS can reduce the brittleness of PLA, resulting in a decrease in the Young’s modulus and ultimate tensile strength. They found that PBS acts as a nucleating agent, increasing the degree of crystallinity of PLA. Further, Zhang et al. [14] reported that 80 wt% PBS in PLA/PBS blends results in a synergistic toughening and strengthening effect, yielding improved tensile properties. Among biodegradable polyesters, PBAT (Figure 1b) is a suitable polymer for blending with PBS. Indeed, PBAT is an aromatic–aliphatic copolyester, produced from oil-based resources, which exhibits good biodegradability in the presence of natural micro-organisms [15]. PBAT is very tough and ductile, can be processed by conventional film extrusion techniques, yielding films with mechanical properties similar to those of LDPE. Due to its random structure, it shows low crystallinity, presenting lower modulus and stiffness when compared to its homopolymers. PBAT incorporation can improve the toughness of polymer blends that contain brittle polymers, such as PLA or thermoplastic starch (TPS), without interfering with their biodegradability [16,17,18,19,20,21]. Concerning gas barrier properties, among biodegradable polymers PBAT shows slightly higher *P*_O_ and water vapor permeability (*P_WV_*) compared to PLA and PBS [5]. Blending PBAT with PLA results in a slight increase in gas permeability with respect to pure PBAT, likely due to the low compatibility of the two polymers [17]. Nonetheless, the higher barrier to oxygen with respect to the polyolefins highlights the potential of these biopolymers and their blends as packaging materials for oxygen-sensitive products [5].

In this framework, although reports on PBS and PBAT compounded with other biodegradable polymers [8,12,17,18] are numerous, studies on PBS/PBAT binary blends are scarce [22,23,24] and their scope is limited, as they have been only focused on injection molded samples. However, it has been reported that the tensile strength of PBS/PBAT blends can be higher than each of the blended partner. Therefore, it is expected that the combination of the high ductility of PBAT with the tensile strength of PBS result in balanced properties for the production of biodegradable films [25]. To this end, in this work blends of PBAT and PBS were prepared and analyzed to investigate how different polymer weight ratios affect blend compatibility, polymer melt rheology, tensile properties, and water vapor and oxygen barriers, aimed at the preparation of biodegradable films for packaging applications.

## 2. Experimental

### 2.1. Materials

The poly(butylene succinate) (PBS, *M*_w_ = 55,000 g/mol, *M*_w_/*M*_n_ = 2.47) used in this work was a non-commercial product supplied by Novamont (Novara, Italy). Poly(butylene adipate-*co*-terephthalate) (PBAT, *M*_w_ = 53,900 g/mol, *M*_w_/*M*_n_ = 1.70) is a commercial product produced by BASF (Ludwigshafen, Germany) under the trade name Ecoflex F-blend C1200. PBS and PBAT polymer pellets were processed in a twin-screw Haake MiniLab extruder (Thermo Scientific, Karlsruhe, Germany), operating at 160 °C and 25 rpm. The strands were pelletized and compression molded using a Carver press operating at 150 °C, adopting the following procedure: 0.5 tons for 3 min, 2 tons for 2 min, and water cooling at 50 °C for 10 min (in order to get films about 200 μm thick). Five sample formulations were thus prepared: pure PBS, pure PBAT, and three PBS/PBAT binary blends, containing 25%, 50% and 75% PBS (*w/w*), coded PBS25/PBAT75, PBS50/PBAT50 and PBS25/PBAT75, respectively.

### 2.2. Characterization Methods

The rheological analysis of neat PBS and PBAT and their blends was performed in the molten state using a strain-controlled ARES (TA) rotational rheometer (TA Instruments, New Castle, DE, USA) equipped with parallel plate geometry (25 mm diameter, 1 mm gap). First, strain sweep tests at the frequency of 1 rad/s were performed to determine the linear viscoelastic region. Small amplitude oscillatory shear measurements, within the linear viscoelasticity regime, were then carried out in the frequency range between 0.1 and 10^2^ rad/s at the temperature of 160 °C, and the shear complex viscosity (η*), the storage (*G*’) and the loss (*G*’’) moduli were measured.

Differential Scanning Calorimetry (DSC) characterization was carried out on compression molded films by a TA Q2000 calorimeter (TA Instruments, New Castle, DE, USA). The following temperature program was employed: heating from 25 to 200 °C, cooling from 200 to −80 °C, further heating from −80 to 250 °C, with a constant heating rate of 10 °C/min. Energy flux versus time data, related to the crystallization peak at the cooling stage, were numerically integrated with a custom software that delivered the crystallization parameters. The specific heat of phase change, Δ*H*, was computed from the total energy transferred, *E*_0_ (from the sample to the environment during the exothermic crystallization process and vice versa during the endothermic melting process).

Mass crystallinity changes were estimated as follows:

(1) $$\Delta X=\frac{\Delta H}{\Delta H_{m}^{o}}=\frac{E_{0}}{m_{S}w_{p}\Delta H_{m}^{o}}$$

where *m*_S_ is the sample mass, *w*_p_ is the mass fraction of the component in the blend, and Δ*H*°_m_ is the specific latent heat of the melting of the 100% crystalline polymer. For the neat resins, values of 110 and 114 J/g for PBS and PBAT, respectively, were taken from the literature [26,27].

Fourier-transform infrared (FTIR) spectroscopy was performed on thin polymer films obtained by casting a drop of polymer solution in chloroform on a KBr disc. Transmission spectra were acquired using a Perkin Elmer Spectrum 100 spectrophotometer (Perkin Elmer, Milan, Italy) operating from 4000 to 400 cm^−1^, performing 16 scans at 1 cm^−1^ resolution.

The morphological analysis of the compression molded films was conducted on a FEI Quanta 200 FEG Scanning Electron Microscope (SEM) (FEI, Eindhoven, Netherlands) on cryogenically fractured cross-sections. SEM observations were performed in low vacuum mode (*p* < 0.7 Torr), using a large field detector (LFD) and an acceleration voltage of 5–20 kV. Selective dissolution of PBAT in tetrahydrofuran (THF) was accomplished to distinguish the polymer phases in the blends. All samples were dried prior to the observation, and their surfaces were sputter coated with a homogeneous layer of Au–Pd alloy to make them conductive, by means of MED020 unit (Baltec AG. Pfäffikon, Switzerland).

The thermal stability of the compression molded films was studied by thermogravimetric analysis (TGA), carried out on a TG-SDTA 851 thermobalance (Mettler-Toledo, Milan, Italy). The measurements were performed on samples of approximately 10 mg, which were placed in ceramic crucibles and heated from 25 to 600 °C at a rate of 20 °C/min, in a nitrogen atmosphere, with a nominal gas flow rate of 30 mL/min.

Tensile tests were performed at 23 ± 2 °C and 45 ± 5% RH on dumbbell-shaped specimens (4 × 28 × 0.25 mm^3^) using an Instron 4505 dynamometer equipped with a load cell of 1 kN, at a deformation rate of 5 mm/min. Prior to the test, the films were conditioned at 25 °C and 50% RH for 48 h. The reported data are the average values of seven determinations.

The water vapor and oxygen permeability of the compression molded films were determined on a Multiperm/PermeO_2_ apparatus (ExtraSolution, Lucca, Italy). The exposed area of the film was 50 cm^2^. Triplicate measurements were performed at 25 °C and 50% RH. Depending on the gas used, water vapor transmission rate (WVTR) (g/(m^2^ × 24 h) or oxygen transmission rate (OTR) (cm^3^(STP)/(m^2^ × 24 h)), where STP stands for standard temperature and pressure, were measured. Water vapor permeability (*P*_WV_) was calculated from WVTR data, using the equation:

(2) $$P_{\mathrm{WV}}=WVTR\frac{h}{\Delta p}$$

where *h* is the film thickness, and Δ*p* is the water partial pressure difference between inner and outer sides of the film, identified with the vapor pressure of water at 25 °C and 50% RH, Δ*p* = 1.58 × 10^3^ Pa.

Oxygen permeability (*P*_O_) was calculated from OTR data, using the equation:

(3) $$P_{O}=\mathrm{OTR}\frac{h}{\Delta p}$$

with Δ*p* = 1, and atm = 1.01 × 10^5^ Pa.

The permeability values were converted to SI units, (mol/m·s·Pa), considering the molar mass of water, 18.02 g/mol, and the normal molar volume of ideal gases, 22,414 cm^3^ (STP)/mol.

## 3. Results and Discussion

### 3.1. Rheological Properties of the Melts

**Figure 2:**
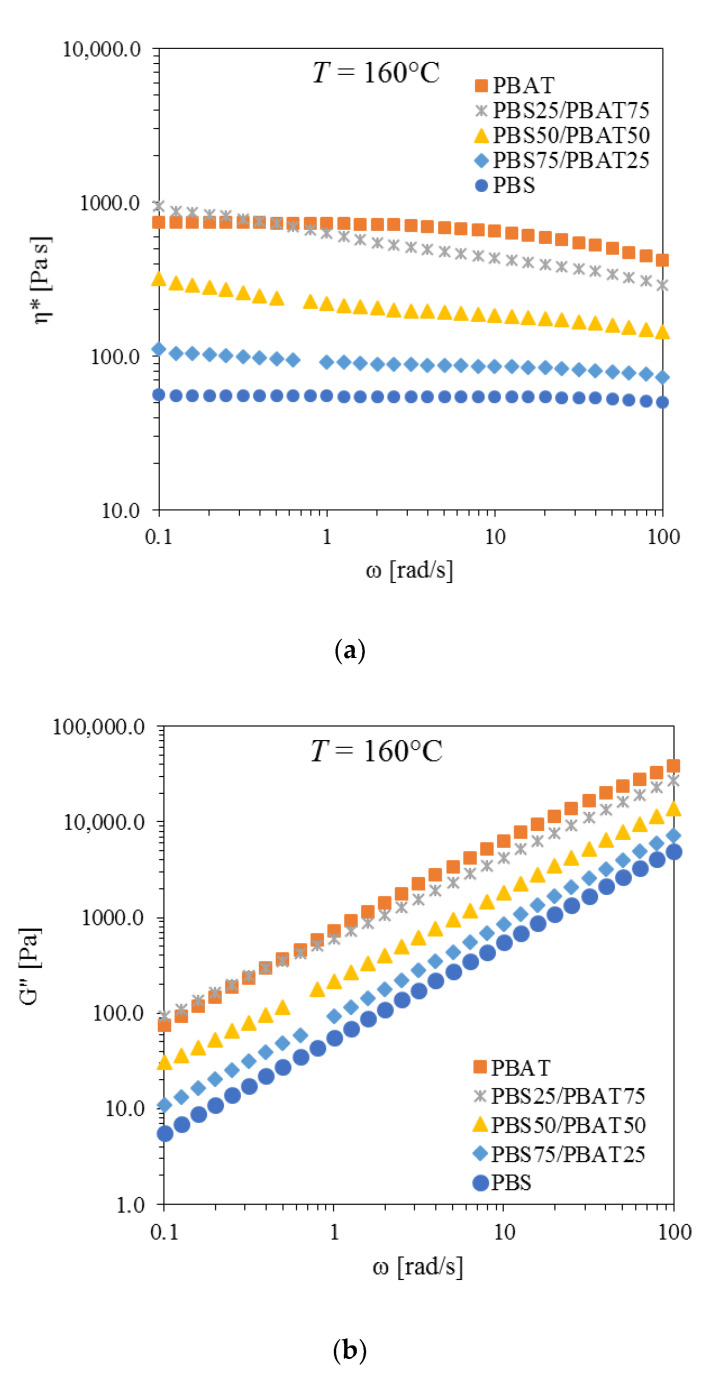

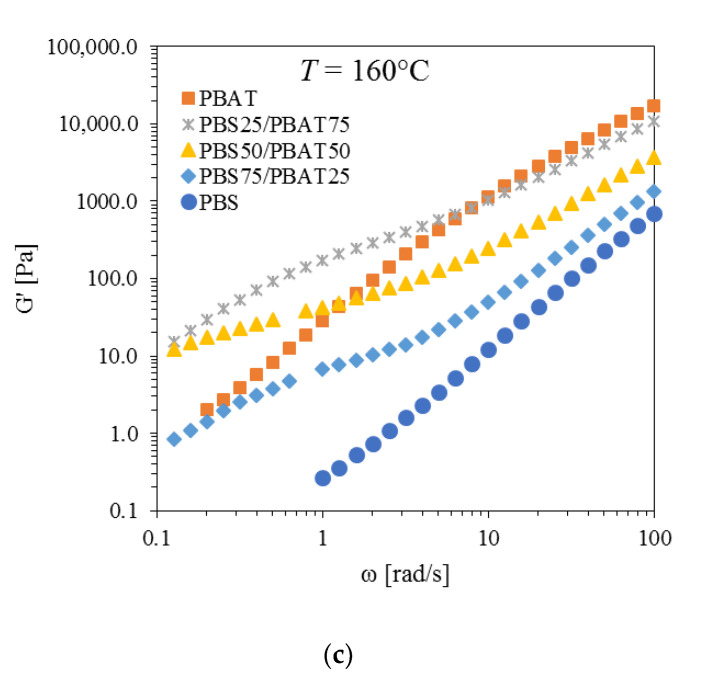
Rheological behavior of PBS, PBAT and their blends at *T* = 160 °C: (**a**) complex viscosity, η*, (**b**) loss modulus, *G*’’, and (**c**) storage modulus, *G*’.

The rheological characterization was performed in the molten state (160 °C) on neat PBS and PBAT, and their blends (Figure 2). Oscillatory shear measurements in the frequency domain were carried out at strains within the linear viscoelastic range (γ_c_ > 100% both for PBS and PBAT). The complex viscosity of neat PBS (Figure S1a) was characteristic of Newtonian behavior in the whole frequency range tested (η_0_ = 80 Pa·s). The examination of *G*’ and *G*’’ curves indicates that at low frequencies, *G*’ tends to zero, as the polymer melt behaves as a viscous liquid. A terminal behavior, with *G*′~ω^2^ and *G*′′~ω, was noticed at lower frequencies [14,15], while at higher frequencies *G*′′ was still prevailing over G′, even at 100 rad/s. Neat PBAT showed a more distinct shear thinning behavior at higher frequencies, and a Newtonian behavior at the lower frequencies (Figure S1b) compared to PBS. Additionally, PBAT displayed increased viscoelastic properties, with viscosity values about two orders of magnitude higher than those of the neat PBS (η_0_ = 1190 Pa·s). Similar to PBS, PBAT did not show any crossover between *G*′′ and *G*′ curves until 100 rad/s.

The results of the rheological characterization of the blends are reported in Figure 2. For both the PBS25/PBAT75 and the PBS50/PBAT50 blends, the complex viscosity displayed a shear thinning behavior at the lower frequencies investigated, which was much less evident in the case of the PBS75/PBAT25 blend (Figure 2a). Moreover, the viscosity of the blends increased with the increasing PBAT contents. In particular, the viscosity curve of the PBS25/PBAT75 blend partly overlapped that of PBAT.

As regards the *G*’’ of the blends, its trend was comparable to that of the pure polymers (Figure 2b). In particular, the blend with the highest content of PBAT (PBS25/PBAT75) displayed slightly higher values in the lower frequency region compared to PBAT, while the curves of the other blends were in between those of pure polymers. Increasing the amount of PBAT in the blends resulted in progressively higher *G*’’ values. As for the elastic modulus (Figure 2c), while PBS and PBAT showed the typical behavior of linear flexible polymers (with terminal behavior and G’ scaling at about ω^2^), indicating that both were fully relaxed at low frequencies, the PBS/PBAT blends were characterized by an enhanced elastic response in the low frequency region. Indeed, a dramatic enhancement of *G*’ occurred for PBS25/PBAT75 and PBS50/PBAT50 in the low frequency region, while the PBS75/PBAT25 curve overlapped with that of PBAT. Notably, PBS25/PBAT75 and PBS75/PBAT25 exhibited a shoulder at about ω ~ 1 rad/s, due to the shape recovery involving the droplets of the disperse phase. Analogous increases in *G*’ in the 10^−2^–10^−1^ rad/s range have been reported for several biodegradable polymer blends, including PLA/PBS [28], PLA/PBAT [17] and PLA/PBSA [29].

Interestingly, the PBS50/PBAT50 blend tended to plateau at lower oscillation frequencies (*G*’ ~ ω^0.6^), likely due to the formation of a co-continuous/interpenetrated morphology, which determines the complex thermoreological behavior. In summary, the presence of two polymeric phases hindered the macromolecules’ relaxation, resulting in an increase in the elastic response at longer measurement times. On the other hand, at higher frequencies, the blends showed a rheological behavior intermediate between that of the pure polymers, characterized by a gradual increase in *G*’ with the increasing PBAT content in the blends.

### 3.2. Differential Scanning Calorimetry (DSC) of Films

**Figure 3:**
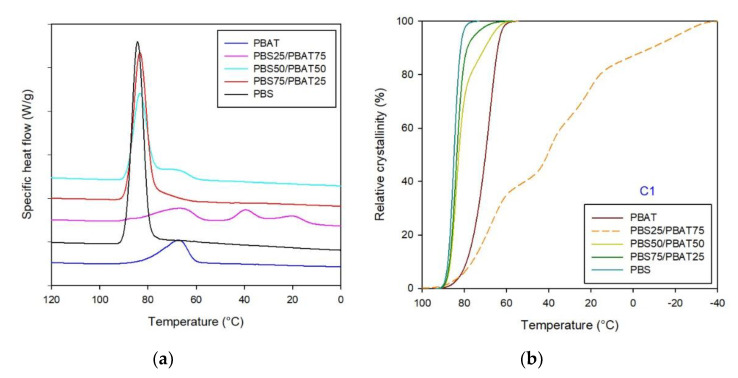
DSC thermograms of PBS, PBAT and their blends: (**a**) cooling from the melt, (**b**) relative crystallinity vs. temperature.

**Figure 4:**
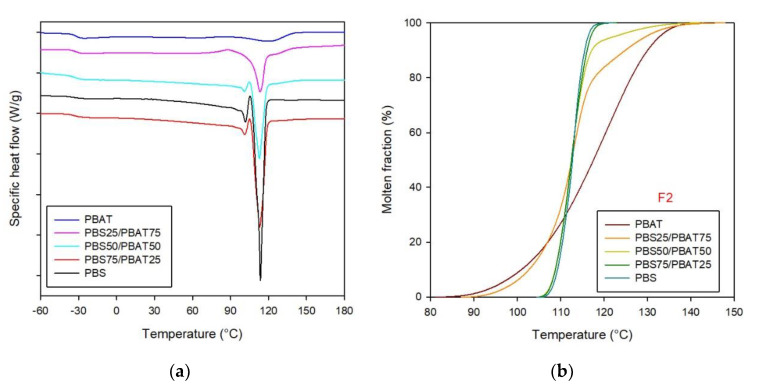
DSC thermograms of PBS, PBAT and their blends: (**a**) second heating run, (**b**) molten fraction vs. temperature.

The thermograms of PBS/PBAT-based films during cooling from melt are reported in Figure 3a, along with the relative crystallinity (Figure 3b) vs. temperature. The calculated numerical results are listed in Table 1, whereas Figure 4 and Table 2 summarize the data relative to the second heating ramp, including DSC thermograms (Figure 4a) and molten fraction (Figure 4b) as a function of temperature.

The cooling step of the neat polymers was characterized by the sharp crystalline peak of PBS and the broad crystallization pattern of PBAT (Figure 3a). Moreover, as expected from a more crystalline polymer, PBS crystallization begun at a higher temperature. As far as the blends are concerned, it is worth highlighting that the PBS/PBAT blends showed multimodal crystallization profiles, corresponding to the different types of crystal lamellae formed during cooling from melt.

From the analysis of the thermograms and the values of the latent heat of crystallization values (Table 1), it was noted that the crystallinity of the blends followed the rule of mixtures (consistent with separate and independent crystallization of the components), dominated by the higher crystallinity components [17]. In particular, the PBS75/PBAT25 and PBS50/PBAT50 blends evidenced a bimodal crystallization pattern; however, the progressive increase in the PBAT fraction resulted in a general modification of the developed PBS crystalline structures, as evidenced by the formation of crystallization peaks that were broader and more shifted towards lower temperatures. This effect was particularly emphasized in the PBS25/PBAT75 formulation, where at least three crystallization phenomena occurred. The first one, at about 67 °C, was attributed to PBAT crystallization, whereas the peaks found at around 40 °C and the less intense one at 21 °C were associated with the crystallization of PBS. By means of peak fitting, it was possible to separate the crystallization peaks of both polymers, as evidenced in Figure S2, and to evaluate their respective crystallization enthalpies (Table 1). Actually, the presence of PBAT in large amounts delayed and slightly inhibited the PBS crystallization process, as evidenced by the normalized crystallization enthalpy values of PBS25/PBAT75 and PBS50/PBAT50. A similar behavior was found for the PBS and poly(propylene carbonate) (PPC) blends, which evidenced that the crystallization temperature of PBS severely decreased with the addition of PPC [30,31].

These results were corroborated by investigating the relative crystallinity as a function of temperature (Figure 3b). From the analysis of the thermal profiles, it is worthy of note that PBS, and the blends with prevailing PBS, evidenced the highest crystallinity and crystallization temperatures, while for the PBS25/PBAT75 blend the crystallization pattern showed small complex peaks, with low crystallinity percentages and peak temperatures even lower than that of neat PBAT. This outcome confirmed the previous findings, highlighting that at the molecular level, PBAT severely modified PBS’s crystallization profile.

DSC curves related to the second heating ramp are shown in Figure 4a. Due to the close proximity of the PBS and PBAT glass transition temperature ranges, their *T*_g_ signals overlapped, and only one value (−32 °C) could be detected from the thermograms for all samples (see Figure 4a and Table 2).

As regards PBS, besides the main melting occurrence, the evidence of cold crystallization, which resulted in the melting, recrystallization and re-melting of PBS crystals during the heating process, was highlighted in all the samples except PBAT. Actually, the cold crystallization process is very common for polyesters [32], and it is strictly correlated with the rate of cooling from the melt. The faster the cooling process, the higher the hindering of melt crystallization. As such, during the second heating ramp, at temperatures above the glass transition, metastable crystalline nuclei form due to the increased macromolecular mobility [33]. In neat PBS, PBS75/PBAT25 and PBS50/PBAT50, the thermograms evidenced two endothermic peaks associated with PBS melting process. The first one at a lower temperature (around 100 °C) was attributed to the melting of cold crystallized shaky crystalline fractions, while the main melting phenomenon, occurring at about 113 °C, was associated with the melting of PBS stable crystals formed during the cooling process from the melt. Similar results were found by Ray et al. [34]. In PBS25/PBAT75, the melting thermogram of the PBS fraction significantly changed. Actually, PBAT amorphous segments, as previously discussed, hindered PBS crystallization from the melt, whilst providing a broad cold crystallization profile starting at around 80 °C. A similar result was found by Arruda et al. for PBAT-PLA blends, in which PBAT reduced PLA’s cold crystallization temperature by about 20 °C [18].

In addition, in the plain PBAT, PBS50/PBAT50 and PBS25/PBAT75 samples, a broad and weak peak was detected, related to the PBAT melting process. In particular, with respect to neat PBAT *T*_m_, occurring at about 120 °C, the blends showed a slight shift of the PBAT melting process towards higher temperatures, and a significant decrease in melting enthalpy and crystallinity, as a consequence of physical interactions occurring between the two polymers.

The molten fraction curves (Figure 4b) showed a sigmoidal shape characteristic of continuous phase transition. Deviations from the sigmoidal trend were observed for PBS50/PBAT50 and PBS25/PBAT75, because of the bimodal and more complex melting behavior of PBS in the two blends. The PBAT melting rate increased with PBS addition, indicating a faster process. This outcome also emphasized that, although the PBS and PBAT melting phenomena occurred separately due to their molecular immiscibility, the physical interaction influencing the reciprocal thermal behavior could be assessed, as also confirmed by the infrared spectroscopy and thermogravimetric analysis reported in the following. It is also worth noting that when PBS is largely prevailing over PBAT, as in PBS75/PBAT25, polymer phase separation is more marked, as demonstrated by the *T*_0.1%_, *T*_99.9%_ and Δ*X*_PBS_ values, which did not change with respect to those of neat PBS.

### 3.3. FTIR Spectroscopy of Films

**Figure 5:**
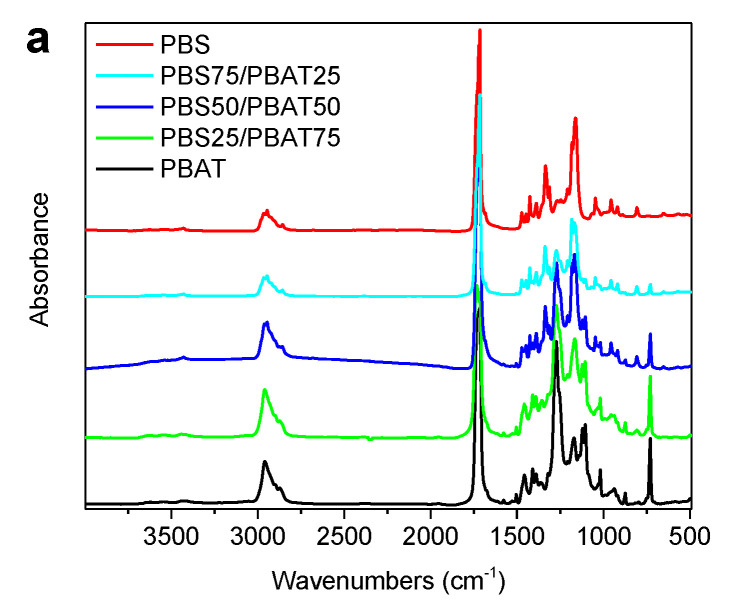

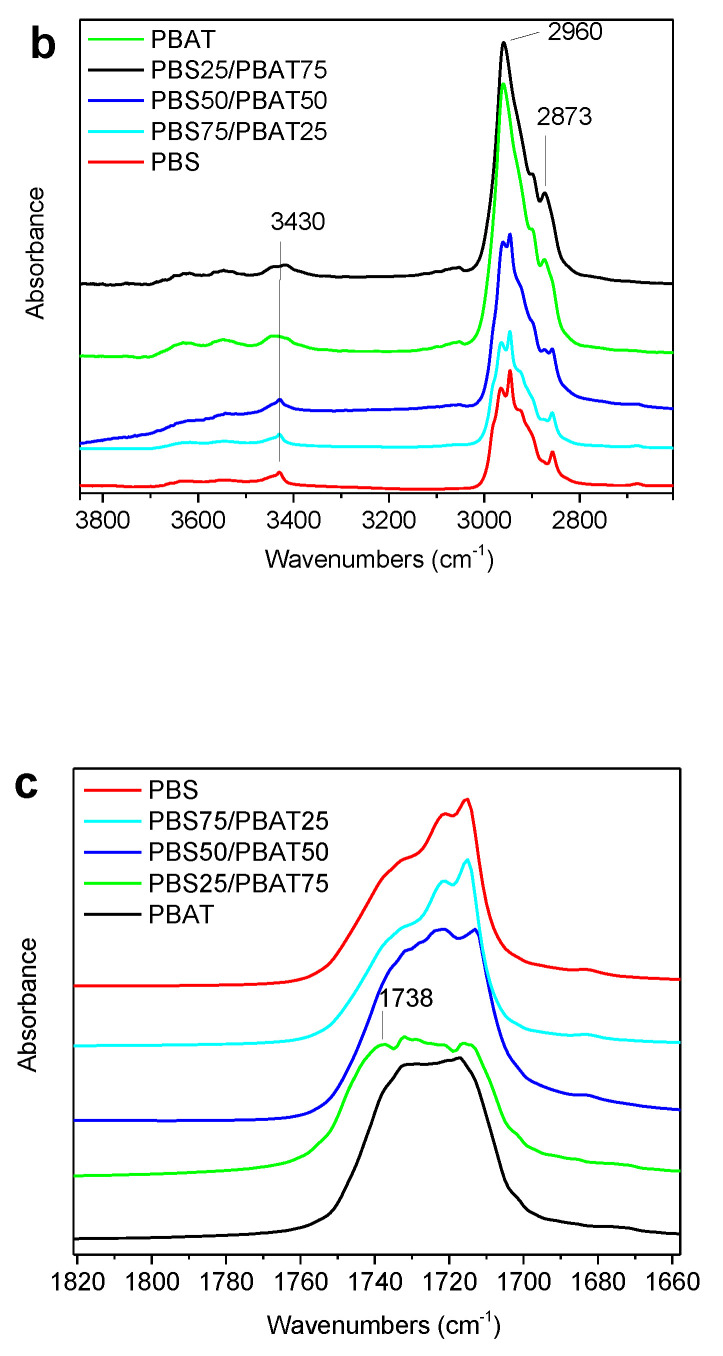
Infrared spectra of neat PBS, PBAT and their blends: (**a**) full spectral region, close-up view of the (**b**) C–H, and (**c**) C=O stretching regions.

Infrared spectroscopy was used to characterize the main functional groups of the neat polyesters, as well as to obtain information related to the possible interactions occurring between them after the melt blending process. The FTIR spectra of PBAT, PBS and their blends are shown in Figure 5a. A close-up view of the C–H and C=O stretching regions are reported in Figure 5b,c, respectively.

As concerns the PBS spectrum, the following functional groups could be evidenced: in the region between 3600 and 3000 cm^−1^ a very weak absorption band of around 3430 cm^−1^ was assigned to the PBS chain terminal hydroxyl groups, while in the range 3000−2800 cm^−1^, the asymmetric stretching of the −CH− groups was identified (the corresponding symmetrical vibration could be found at 1335 cm^−^^1^).

As usually evidenced in semicrystalline polyesters, the carbonyl group region (−C=O) was made up of several overlapping peaks. Previous studies showed that these peaks arise from the stretching of the amorphous and crystalline domains of ester carbonyl groups [35].

In plain PBS, the first broad peak, corresponding to the stretching associated with the amorphous fraction of the polymer, occurred in the 1720−1730 cm^−1^ range, as a shoulder of the sharper and more intense band at about 1711 cm^−1^, imputable to the carbonyl stretching of the crystalline domains of the polymer [36]. The peaks at about 1160 cm^−1^ and 1210 cm^−1^ corresponded to the −C–O–C– stretching of the ester bonds, while the absorption band at about 1044 cm^−1^ was ascribed to the –O–C–C– stretching vibration. Finally, the peak at 956 cm^−1^ corresponded to the bending of the –C–OH of the terminal acid groups. The main absorption peaks of PBAT, assigned to the asymmetric and symmetric stretching vibrations of the CH_2_ groups, occurred at 2960 and 2873 cm^−1^, respectively. A more detailed analysis of the PBAT infrared spectrum in the carbonyl region highlighted the presence of a strongly convoluted C=O absorption band between 1700 and 1740 cm^−1^, due to different chemical surroundings, including intra- and inter-molecular hydrogen bonding, resulting in manifold stretching vibration energies. The main peaks in this region were centered at 1732 and 1715 cm^−1^. In particular, the peak at 1732 cm^−1^ has been attributed to carbonyl groups in the amorphous polymer fraction [37], while the peak at 1715 cm^-1^ could be associated with site-specific interactions, occurring given the conjugative effect between the phenylene aromatic groups and the carbonyl groups of PBAT terephthalate residues [10,38].

The characteristic stretching peaks of the C=C phenylene group occurred at 1505 and 1459 cm^−1^. The peaks at 1410 and 1390 cm^−1^ corresponded to trans–CH_2_–plane bending vibrations [39], while those at 1268 cm^−1^ were assigned to C–O asymmetric stretching vibrations. The peaks at 873 and 727 cm^−1^ represented the out-of-plane bending vibration of the phenylene ring [39].

It is worth mentioning that in neat PBAT, the absorption corresponding to the symmetric stretching of the C–O bond for the adipate and terephthalate fractions appears as a broad and intense peak at 1172 cm^−1^, and as a sharp and smaller peak at 1110 cm^−1^, respectively. This outcome suggested that in the PBAT used, there was a higher concentration of PBA aliphatic amorphous segments. This hypothesis was confirmed by the mechanical performances of the films discussed in the following. Similar results have been found by Herrera et al. [40].

As concerns the blends, a more complex pattern of carbonyl group vibration modes could be observed. Indeed, by increasing the percentage of PBAT, the amorphous component of the C=O stretching vibration was ever more pronounced and shifted towards higher frequency (1738 cm^−1^). These results are consistent with the previous findings related to the higher concentration of flexible PBA macromolecular chains, and match well with DSC properties previously discussed [41].

### 3.4. Scanning Electron Microscopy (SEM) of Films

**Figure 6:**
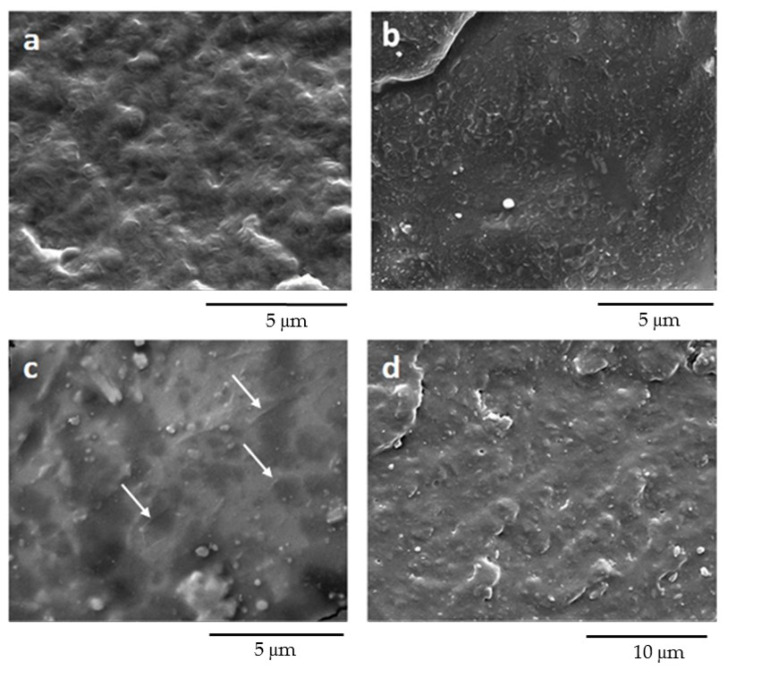
SEM micrographs of (**a**) PBAT, (**b**) PBS, (**c**) PBS25/PBAT75 and (**d**) PBS75/PBAT25.

SEM pictures of the cross-sections of cryogenically fractured films of PBAT, PBS and their blends are shown in Figure 6. The micrograph of neat PBAT (Figure 6a) evidenced a textured surface, characterized by the presence of several roughly round micro-structured domains, reflecting the complex and heterogeneous structural arrangement of PBAT, as well as its rather ductile behavior upon fracture [42]. In Figure 6b, the cross-sectional fracture of plain PBS highlighted a smoother structure with a large fracture plane, which suggests a predominantly brittle cryogenic fracture, although the presence of ridges accounts for the partial tenacity of the polymer. Several irregularly sized rough edges, likely associated with the presence of PBS crystalline particles, could also be detected. All the blends were generally characterized by smoother surfaces where both phases co-exist with a droplet-matrix or co-continuous phase morphology. Nonetheless, a clear interface between the phases could not be noticed due to the above-discussed interaction between the two polymers. In the PBS25/PBAT75 blend (Figure 6c), it was possible to observe an inhomogeneous morphology, in which the isolated domains of PBS, featuring sharp contours (highlighted by white arrows), were evenly dispersed in the ductile PBAT matrix. In PBS75/PBAT25 (Figure 6d), well-defined PBS crystalline particles appeared; regardless, the presence of a more ductile PBAT dispersed phase induced a change in morphology, from a rigid surface to a smoother one featuring small fracture planes. These morphologies suggested a good macromolecular interconnection between the two polymeric phases. Similar results have been reported by Gigante et al. [43].

**Figure 7:**
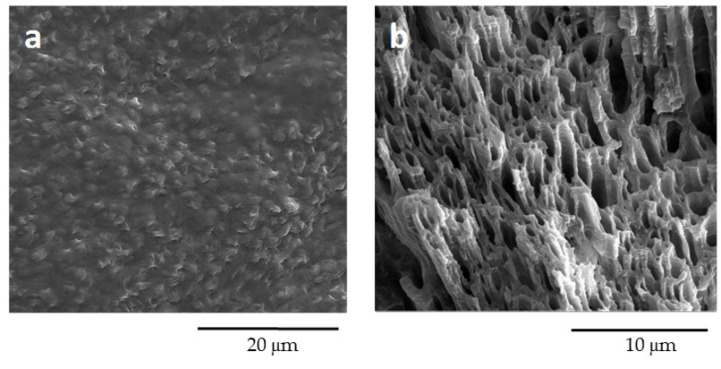
SEM images of PBS50/PBAT50: (**a**) film surface and (**b**) cryogenic fracture surface after PBAT removal by etching in THF.

In order to better highlight the structure and morphology of the two phases, the PBS50/PBAT50 film was soaked in tetrahydrofuran (THF), a solvent able to selectively dissolve PBAT. Both the film surface and cryogenic fracture surface exposed to THF are visualized in Figure 7.

The PBAT dissolved after 5 min of treatment, leaving the PBS structure clean and intact. The observation of the film surface exposed to THF confirmed the effective removal of the overlying amorphous PBAT layer, revealing the presence of regularly structured PBS crystallites. Indeed, after the etching process, cylindrical channels surrounded by PBS crystalline polymer chains were observed, demonstrating the formation of two submicron co-continuous phases. It worth underlining that the PBS crystallites were arranged perpendicularly to the direction of load application during the compression molding. These morphologies were typical of immiscible polymers with high physical compatibility [44], corroborating the above-discussed DSC results.

### 3.5. Thermogravimetric Analysis (TGA) of Films

**Figure 8:**
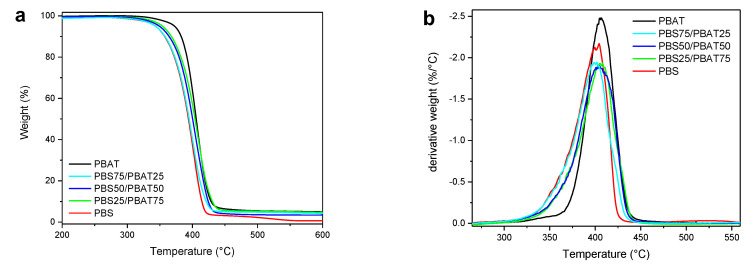
(**a**) Thermogravimetric (TGA) curves and (**b**) derivative (DTG) TGA curves of PBAT, PBS and their blends under nitrogen.

In Figure 8, TGA and the derivative curve profiles of the PBS/PBAT-based films are reported, whereas the thermal parameter results are summarized in Table 3. Degradation of all samples occurred in a single major weight loss step, although PBS also showed a minor weight loss step above 500 °C. From the analysis of the temperatures at which 5% weight loss occcured (*T*_5%_), it is noticeable that PBAT is more thermally stable than PBS. As for the blends, even the presence of a low amount of PBS impaired the thermal stability of PBAT, as demonstrated by the significant decrease in *T*_5%_ detected for PBS25/PBAT75 compared to plain PBAT. Further decreasing the PBAT content in the blends caused a gradual reduction in *T*_5%_ all the way down to that of PBS (343 °C). PBAT also yielded the highest residue at 600 °C, likely due to its aromatic fraction, which is able to form a stable char upon pyrolysis. It was also noted that PBAT could slightly increase the char yield in the blends compared to PBS. From the analysis of the DTG thermograms, the temperatures of maximum degradation rate (*T*_max_) were calculated, as listed in Table 3. A single degradation peak was noticed, and its temperature was comparable for all samples. That is, it is likely that the initiation of PBS degradation also involves the PBAT phase, confirming the partial physical compatibility between the two polymers.

### 3.6. Mechanical Properties of Films

Tensile tests were carried out on the compression molded films, and the results obtained are listed in Table 4, while illustrative stress–strain curves are reported in Figure S3. From the analysis of the data, it is noticeable that the neat PBS used in the present work was rather stiff, evidencing a high elastic modulus, relatively high tensile strength and a very low elongation at break. Typically, commercially available PBS, unlike the polymer used in this work, is made more ductile by post-synthesis chain extension reactions. In fact, non-commercial PBS has similar properties to those reported in Table 4. As an example, Tan et al. [45] synthesized a PBS with a molecular weight *M*_w_ = 58 kg/mol (*M*_w_ of the PBS used in this work = 62 kg/mol), which exhibited values of elastic modulus and elongation at break equal to 607 MPa and 10.2%, respectively. Conversely, post-synthesis-modified commercial PBS has significantly higher molecular weight values; for example, the Bionolle 1020 made by Showa Denko has an *M*_w_ = 140 kg/mol [23].

On the other hand, neat PBAT was very ductile, with a low elastic modulus and a very high elongation at break. PBAT’s mechanical properties are comparable to those of low-density PE (LDPE), making it a promising biodegradable material for a wide range of potential applications, from medical devices to agricultural and packaging films [15,26]. In addition, it has been reported that the mechanical properties of PBAT strongly depend on the PBT and PBA fraction contents. Indeed, the elongation at break remarkably increases as the terephthalate aromatic segments decrease [39,46]. As expected, the addition of PBS to PBAT led to blends with increased modulus and decreased elongation at break; that is, blend rigidity increased with PBS content. In particular, contents of PBS higher than 25% by weight dramatically decreased the blend’s ductility. The dependence of mechanical properties on composition is expected for blends of a crystalline polymer (PBS) with a more amorphous one (PBAT). Indeed, in the presence of large amounts of the crystalline PBS, phase separation occurs to a greater extent, as demonstrated by the DSC results, dramatically affecting the mechanical properties. As a consequence, ductility and tenacity drop down, as shown by PBS75/PBAT25. Nevertheless, a suitable compromise between stiffness and ductility (*E* = 135 MPa, ε = 390%) was found for the PBS25/PBAT75 blend, as in this case the mechanical performance was likely influenced by the partial compatibility between the two polymers in the amorphous phase.

### 3.7. Water Vapor and Oxygen Permeability of Films

The oxygen and water vapor permeability results, measured at 25 °C for the neat PBAT and PBS films and their blends, are shown in Table 5.

The *P*_WV_ values of neat PBS and PBAT films were 2.74 × 10^−12^ and 7.09 × 10^−12^ mol/m s Pa, respectively, which were slightly higher than those reported in the literature, in particular concerning PBAT, for which *P*_WV_ values comparable to those of PBS have been reported [5]. This difference may be ascribed to the very low crystallinity of the PBAT grade used in the present work. As for the blends, *P*_WV_ was expected to decrease with increasing PBS contents. Indeed, the *P*_WV_ values of the PBS25/PBAT75, PBS50/PBAT50 and PBS75/PBAT25 blends decreased by approximately 9%, 46% and 60% with increasing PBS contents. The expected decreases estimated by the rule of mixtures for these same blends were 17%, 31% and 46%, respectively. Thus, the values obtained for PBS50/PBAT50 and PBS75/PBAT25 were smaller to those predicted by the rule of mixtures, suggesting a good interaction between the two polymer phases.

The *P*_O_ values of the PBS and PBAT films were 0.98 × 10^−16^ and 4.41 × 10^−16^ mol/m·s·Pa, respectively. Further, for *P*_O_, its values in the PBS/PBAT blends decreased with PBS content, and the values obtained for all blends were smaller than those predicted by the rule of mixtures. In particular, the permeability value of PBS75/PBAT25 is less than one order of magnitude higher than that reported for the PLA/PBAT composite films obtained by constructing an anisotropic network of ductile PBAT nanofibrils dispersed in an oriented PLA crystalline structure, which requires a far more complex processing approach [47]. These data indicate that both the water vapor and gas barrier properties of PBS films can be tuned by PBAT blending. The favorable effect of PBS on gas permeability has been reported for blends of PBS with cellulose triacetate (CTA) [48] and poly(lactic acid) (PLA) [11], and it is attributed to the microstructural changes caused by blending. In the present work, DSC showed the crystallinity increasing with PBS content in the blends, and consequently the barrier properties should decrease with PBS content, as was actually observed. These results are consistent with the structure of the materials: the higher crystallinity of PBS with respect to PBAT leads to a more tortuous path within the amorphous phase, and reduces the diffusivity of the permeants [9].

## 4. Conclusions

In this paper, blends of PBS and PBAT were prepared at different weight ratios, to produce biodegradable films with tailored physical properties.

PBS and PBAT exhibited a Newtonian behavior in a dynamic regime at low frequencies, while the binary blends exhibited a marked shear thinning behavior. The crystallization of both PBS and PBAT was inhibited in the blends, while the melting points of the compounds were virtually independent of composition. The melt crystallization temperature and rate of crystallization increased with PBS content. FTIR and SEM analysis suggested that a limited interaction exists between the two polymer phases. The mechanical characterization of the blends demonstrated that PBAT, which is a highly ductile and low-crystalline polymer, acts as a plasticizer in PBAT/PBS blends. As PBAT content increases, (i) elongation at break increases, and (ii) the surface fracture becomes more textured, indicating the presence of a more complex energy dissipation mechanism, due to the overall increased content of amorphous phase, which is responsible for a predominantly ductile fracture behavior. In particular, the PBS25/PBAT75 blend is characterized by an enhanced elastic modulus compared to that of PBAT, and by a high elongation at break compared to neat PBS, while the blends with higher PBS content are more brittle. The gas barrier properties tended to decrease with PBS content, and the values obtained for all blends were smaller than those predicted by the rule of mixtures.

Overall, the products based on these blends provide great potential, since their mechanical properties, such as ductility, stiffness and tensile strength, viscosity and barrier properties, are still suitable for various applications, such as industrial packaging.

## Figures and Tables

**Table 1:** DSC results for PBS, PBAT and their blends during the cooling from melt. The values of crystallization enthalpy in parentheses are normalized with respect to the weight fraction of the polymer in the blends.

Sample	*T*_0.1%_ (°C)	*T*_99%_ (°C)	*T*_c PBAT_ (°C)	*T*_c PBS_ (°C)	Δ*H*_c PBAT_ (J/g)	Δ*H*_c PBS_ (J/g)
PBAT	91.4	56.8	67.4	-	21.0	-
PBS25-PBAT75-	99.3	−38	66.6	40.0 (*T*_c1_), 21.3 (*T*_c2_)	17.5 (23.3)	15.6 (62.4)
PBS50/PBAT50	91.2	58.3	69.2	83.3	9.5 (19)	33.4 (66.8)
PBS75/PBAT25	91.4	62.4	N.D.	83.3	6.1 (24.4)	52.5 (70)
PBS	91.8	75.2	-	84.3	-	72.3

*T*_0.1%_ and *T*_99.9%_ are the temperatures required to achieve 0.1% and 99.9% crystalline fraction, *T*_c_ is the crystallization peak temperature and Δ*H*_c_ is the crystallization enthalpy.

**Table 2:** DSC results for PBS, PBAT and their blend during the second heating. The values of melting enthalpy in parentheses are normalized with respect to the weight fraction of the polymer in the blends.

Sample	*T*_0.1%_ (°C)	*T*_99%_ (°C)	*T*_m PBAT_ (°C)	*T_m PBS_* (°C)	Δ*H*_m PBAT_ (J/g)	Δ*H*_m PBS_ (J/g)	Δ*X*_PBAT_ (%)	Δ*X*_PBS_ (%)
PBAT	84.3	140.2	120.9	-	22.2	-	19.5	-
PBS25/PBAT75	89.7	143.6	122.5	114.0	8.3 (11.1)	16.7 (66.8)	9.7	60.7
PBS50/PBAT50	90.8	133.8	122.8	101.1 (*T*_m1_), 113.1 (*T*_m2_)	3.9 (7.8)	28.4 (56.8)	6.8	51.6
PBS75/PBAT25	105.4	120.3	N.D.	101.2 (*T*_m1_), 113.0 (*T*_m2_)	1.9 (7.6)	54.1 (72.1)	6.7	65.6
PBS	106.0	119.4	-	101.8 (*T*_m1_), 113.6 (*T*_m2_)	-	72.2	-	65.6

*T*_0.1%_ and *T*_99.9%_ are the temperatures required to achieve 0.1% and 99.9% molten fraction or relative crystallinity, *T*_m_ is the melting peak temperature, Δ*H*_m_ is the melting enthalpy, and ΔX is the change in crystallinity during the event.

**Table 3:** Thermal parameters of PBAT, PBS and their blends from TGA.

Sample	*T*_5%_ (°C)	*T*_max_ (°C)	Char_600_ (%)
PBAT	373	407	5.0
PBS25/PBAT75	354	406	4.7
PBS50/PBAT50	349	404	3.4
PBS75/PBAT25	343	400	4.2
PBS	343	402	0.6

**Table 4:** Tensile properties of PBAT, PBS and their blends.

Sample	Elastic Modulus *E*	Strain at Break ε	Stress at Break σ_R_
	MPa	%	MPa
PBAT	81.0 ± 2.5	689.5 ± 110.3	20.1 ± 2.4
PBS25/PBAT75	134.9 ± 1.1	393.1 ± 13.0	22.7 ± 1.1
PBS50/PBAT50	299.8 ± 4.0	16.4 ± 0.3	15.2 ± 0.0
PBS75/PBAT25	488.8 ± 7.2	10.2 ± 0.3	10.2 ± 0.2
PBS	725.6 ± 15.6	7.1 ± 0.3	29.6 ± 1.5

**Table 5:** Water vapor and oxygen gas permeabilities of PBAT, PBS and their blends.

Samples	Water Vapor	Oxygen
	*P*_WV_ × 10^12^ [mol/m·s·Pa]	*P*_O_ × 10^16^ [mol/m·s·Pa]
PBAT	7.09 ± 0.08	4.41 ± 0.01
PBS25/PBAT75	6.49 ± 0.03	3.13 ± 0.01
PBS50/PBAT50	3.83 ± 0.04	1.23 ± 0.01
PBS75/PBAT25	2.90 ± 0.02	0.68 ± 0.01
PBS	2.74 ± 0.01	0.98 ± 0.01

## Supplementary Materials

The following are available online at https://www.mdpi.com/2073-4360/12/10/2317/s1, Figure S1: Oscillatory shear measurements in the frequency domain carried out at 160 °C on: (a) PBS, (b) PBAT, Figure S2: DSC crystallization peak deconvolution of PBS50-PBAT50 blend highlighting the PBS (green curve) and PBAT (blue curve) cooling crystallization peaks, Figure S3: Illustrative stress-strain curves of films based on neat PBS, PBAT, and their blends.

## References

[1] Xu J., Guo B.H. (2010). Poly(butylene succinate) and its copolymers: Research, development and industrialization. Biotechnol. J..

[2] Guidotti G., Soccio M., Siracusa V., Gazzano M., Salatelli E., Munari A., Lotti N. (2017). Novel random PBS-based copolymers containing aliphatic side chains for sustainable flexible food packaging. Polymers.

[3] De Santis F., Volpe V., Pantani R. (2017). Effect of molding conditions on crystallization kinetics and mechanical properties of poly(lactic acid). Polym. Eng. Sci..

[4] Scarfato P., di Maio L., Milana M.R., Giamberardini S., di Maio L., Denaro M., Incarnato L. (2017). Performance properties, lactic acid specific migration and swelling by simulant of biodegradable poly(lactic acid)/nanoclay multilayer films for food packaging. Food Addit. Contam. Part A Chem. Anal. Control Expo. Risk Assess.

[5] Jost V. (2018). Packaging related properties of commercially available biopolymers—An overview of the status quo. eXPRESS Polym. Lett..

[6] Coltelli M., Aliotta L., Gigante V., Bellusci M., Cinelli P., Bugnicourt E., Schmid M., Staebler A., Lazzeri A. (2020). Protein Isolate Based Blends. Molecules.

[7] Mallegni N., Phuong T., Coltelli M.-B., Cinelli P., Lazzeri A. (2018). Poly(lactic acid) (PLA) Based Tear Resistant and Biodegradable Flexible Films by Blown Film Extrusion. Materials.

[8] Nobile M.R., Cerruti P., Malinconico M., Pantani R. (2015). Processing and properties of biodegradable compounds based on aliphatic polyesters. J. Appl. Polym. Sci..

[9] Charlon S., Follain N., Soulestin J., Sclavons M., Marais S. (2017). Water transport properties of poly(butylene succinate) and poly[(butylene succinate)-co-(butylene adipate)] Nanocomposite films: Influence of the water-assisted extrusion process. J. Phys. Chem. C.

[10] Yao S.F., Chen X.T., Ye H.M. (2017). Investigation of Structure and Crystallization Behavior of Poly(butylene succinate) by Fourier Transform Infrared Spectroscopy. J. Phys. Chem. B.

[11] Luzi F., Fortunati E., Jiménez A., Puglia D., Pezzolla D., Gigliotti G., Kenny J.M., Chiralt A., Torre L. (2016). Production and characterization of PLA/PBS biodegradable blends reinforced with cellulose nanocrystals extracted from hemp fibres. Ind. Crops Prod..

[12] John J., Mani R., Bhattacharya M. (2002). Evaluation of compatibility and properties of biodegradable polyester blends. J. Polym. Sci. Part A Polym. Chem..

[13] Qiu T.Y., Song M., Zhao L.G. (2016). Testing, characterization and modelling of mechanical behaviour of poly (lactic-acid) and poly (butylene succinate) blends. Mech. Adv. Mater. Mod. Process..

[14] Zhang X., Liu Q., Shi J., Ye H., Zhou Q. (2018). Distinctive Tensile Properties of the Blends of Poly(l-lactic acid) (PLLA) and Poly(butylene succinate) (PBS). J. Polym. Environ..

[15] Tan F.T., Cooper D.G., Marić M., Nicell J.A. (2008). Biodegradation of a synthetic co-polyester by aerobic mesophilic microorganisms. Polym. Degrad. Stab..

[16] Ferreira F.V., Cividanes L.S., Gouveia R.F., Lona L.M.F. (2019). An overview on properties and applications of poly(butylene adipate- *co* -terephthalate)-PBAT based composites. Polym. Eng. Sci..

[17] Dil E.J., Carreau P.J., Favis B.D. (2015). Morphology, miscibility and continuity development in poly(lactic acid)/poly(butylene adipate-co-terephthalate) blends. Polymer (Guildf).

[18] Arruda L.C., Magaton M., Bretas R.E.S., Ueki M.M. (2015). Influence of chain extender on mechanical, thermal and morphological properties of blown films of PLA/PBAT blends. Polym. Test..

[19] Al-Itry R., Lamnawar K., Maazouz A. (2014). Rheological, morphological, and interfacial properties of compatibilized PLA/PBAT blends. Rheol. Acta..

[20] Dong W., Zou B., Yan Y., Ma P., Chen M. (2013). Effect of chain-extenders on the properties and hydrolytic degradation behavior of the poly(lactide)/ poly(butylene adipate-co-terephthalate) blends. Int. J. Mol. Sci..

[21] Dammak M., Fourati Y., Tarrés Q., Delgado-Aguilar M., Mutjé P., Boufi S. (2020). Blends of PBAT with plasticized starch for packaging applications: Mechanical properties, rheological behaviour and biodegradability. Ind. Crops Prod..

[22] Muthuraj R., Misra M., Mohanty A.K. (2017). Biocomposite consisting of miscanthus fiber and biodegradable binary blend matrix: Compatibilization and performance evaluation. RSC Advances.

[23] Muthuraj R., Misra M., Mohanty A.K. (2014). Biodegradable Poly(butylene succinate) and Poly(butylene adipate-co-terephthalate) Blends: Reactive Extrusion and Performance Evaluation. J. Polym. Environ..

[24] Chieng B.W., Ibrahim N.A., Yunus W.M.Z.W. (2010). Effect of organo-modified montmorillonite on poly (butylene succinate)/poly (butylene adipate-co-terephthalate) nanocomposites. Express Polym. Lett..

[25] Nobile M.R., Crocitti A., Malinconico M., Santagata G., Cerruti P. (2018). Preparation and characterization of poly(butylene succinate) (PBS) and polybutylene adipate-terephthalate (PBAT) biodegradable blends. AIP Conf. Proc..

[26] Pérez-Camargo R.A., Fernández-D’Arlas B., Cavallo D., Debuissy T., Pollet E., Avérous L., Müller A.J. (2017). Tailoring the structure, morphology, and crystallization of isodimorphic poly(butylene succinate-ran-butylene adipate) random copolymers by changing composition and thermal history. Macromolecules.

[27] Fukushima K., Wu M.H., Bocchini S., Rasyida A., Yang M.C. (2012). PBAT based nanocomposites for medical and industrial applications. Mater. Sci. Eng. C.

[28] Yokohara T., Yamaguchi M. (2008). Structure and properties for biomass-based polyester blends of PLA and PBS. Eur. Polym. J..

[29] Nofar M., Maani A., Sojoudi H., Heuzey M.C., Carreau P.J. (2015). Interfacial and rheological properties of PLA/PBAT and PLA/PBSA blends and their morphological stability under shear flow. J. Rheol.

[30] Chen R., Zou W., Zhang H., Zhang G., Qu J. Crystallization behavior and thermal stability of poly(butylene succinate)/poly(propylene carbonate) blends prepared by novel vane extruder. Proceedings of the 31st International Conference of the Polymer Processing Society.

[31] Pivsa-Art S., Thumsorn S., Pavasupree S., O-Charoen N., Pivsa-Art W., Yamane H., Ohara H. (2013). Effect of additive on crystallization and mechanical properties of polymer blends of poly(lactic acid) and poly[(butylene succinate)-co-adipate]. Energ. Proc..

[32] Ma P.M., Wang R.Y., Wang S.F., Zhang Y., Zhang Y.X., Hristova D. (2008). Effects of fumed silica on the crystallization behavior and thermal properties of poly(hydroxybutyrate-co-hydroxyvalerate). J. Appl. Polym. Sci..

[33] Song L., Qiu Z. (2009). Crystallization behavior and thermal property of biodegradable poly(butylene succinate)/functional multi-walled carbon nanotubes nanocomposite. Polym. Degrad. Stab..

[34] Ray S.S., Bandyopadhyay J., Bousmina M. (2007). Thermal and thermomechanical properties of poly[(butylene succinate)-co-adipate] nanocomposite. Polym. Degrad. Stab..

[35] Mallardo S., de Vito V., Malinconico M., Volpe M.G., Santagata G., di Lorenzo M.L. (2016). Poly(butylene succinate)-based composites containing β-cyclodextrin/d-limonene inclusion complex. Eur. Polym. J..

[36] Dong T., He Y., Shin K.M., Inoue Y. (2004). Formation and characterization of inclusion complexes of poly(butylene succinate) with α- and γ-cyclodextrins. Macromol. Biosci..

[37] Hexig A., Hexig B. (2012). Characterization of Compositional Gradient Structure of Polymeric Materials by FTIR Technology. Infrared Spectrosc.

[38] Dos Santos Rosa B., Merlini C., Livi S., de Oliviera Barra G.M. (2019). Development of poly (butylene adipate-co-terephthalate) filled with montmorillonite-polypyrrole for pressure sensor applications. Mater. Res..

[39] Tavares L.B., Ito N.M., Salvadori M.C., Santos D.J.d., Rosa D.S. (2018). PBAT/kraft lignin blend in flexible laminated food packaging: Peeling resistance and thermal degradability. Polym. Test..

[40] Herrera R., Franco L., Rodríguez-Galán A., Puiggalí J. (2002). Characterization and degradation behavior of poly(butylene adipate-co-terephthalate)s. J. Polym. Sci. Part A Polym. Chem..

[41] Murphy S.H., Leeke G.A., Jenkins M.J. (2012). A Comparison of the use of FTIR spectroscopy with DSC in the characterisation of melting and crystallisation in polycaprolactone. J. Therm. Anal. Calorim..

[42] Dou Q., Cai J. (2016). Investigation on Polylactide (PLA)/Poly(butylene adipate-co-terephthalate) (PBAT)/Bark Flour of Plane Tree (PF) Eco-Composites. Materials.

[43] Gigante V., Coltelli M.-B., Vannozzi A., Panariello L., Fusco A., Trombi L., Donnarumma G., Danti S., Lazzeri A. (2019). Flat Die Extruded Biocompatible Poly(Lactic Acid) (PLA)/Poly(Butylene Succinate) (PBS) Based Films. Polymers.

[44] Wenyong D., Xu W., Yongjin L. (2018). Formation of co-continuous PLLA/PC blends with significantly improved physical properties by reactive comb polymers. J. Appl. Polym. Sci..

[45] Tan L., Chen Y., Zhou W., Nie H., Li F., He X. (2010). Novel poly(butylene succinate-co-lactic acid) copolyesters: Synthesis, crystallization, and enzymatic degradation. Polym. Degrad. Stab..

[46] Lee S.H., Lim S.W., Lee K.H. (1999). Properties of potentially biodegradable copolyesters of (succinic acid-1,4-butanediol)/(dimethyl terephthalate-1,4-butanediol). Polym. Int..

[47] Zhou S.Y., Huang H.-D., Ji X., Yan D.-X., Zhong G.-J., Hsiao B.S., Li Z.-M. (2016). Super-Robust Polylactide Barrier Films by Building Densely Oriented Lamellae Incorporated with Ductile in Situ Nanofibrils of Poly(butylene adipate-co-terephthalate). ACS Appl. Mater. Interf..

[48] Číhal P., Vopička O., Lanč M., Kludský M., Velas J., Hrdlička Z., Michalcová A., Dendisová M., Friess K. (2018). Poly(butylene succinate)-cellulose triacetate blends: Permeation, pervaporation, sorption and physical structure. Polym. Test..

