# Supplementary material for: Properties of Biodegradable Films Based on Poly(butylene Succinate) (PBS) and Poly(butylene Adipate-co-Terephthalate) (PBAT) Blends †
Source: Polymers (Basel). 2020 Oct 10;12(10):2317. doi: 10.3390/polym12102317 (PMC7600530; doi:10.3390/polym12102317)
Supplement: Supplementary file 1 [file polymers-12-02317-s001.pdf]

# Supporting Information

# Properties of biodegradable films based on poly(butylene succinate) (PBS) and poly(butylene adipate-*co*-terephthalate) (PBAT) blends

**Anna Raffaela de Matos Costa[1], Andrea Crocitti[2], Laura Hecker de Carvalho[3,*], Sabrina Carola Carroccio[4], Pierfrancesco Cerruti[5,*] and Gabriella Santagata[5]**

[1]Department of Chemical Engineering, Federal University of Pernambuco, Recife, Brazil
[2]Department of Industrial Engineering, University of Salerno, Via Giovanni Paolo II, 132, 84084 Fisciano (SA), Italy
[3]Departament of Materials Engineering, Federal University of Campina Grande, Campina Grande, PB, 58429-900, Brazil
[4]Institute for Polymers, Composites and Biomaterials (IPCB-CNR), Via Campi Flegrei 34, I-80078 Pozzuoli, Italy
[5]Institute for Polymers, Composites and Biomaterials (IPCB-CNR), Via Paolo Gaifami 18, 9-95126 Catania, Italy

This paper is dedicated to the memory of Prof. Eduardo Luís Canedo and Maria Rossella Nobile

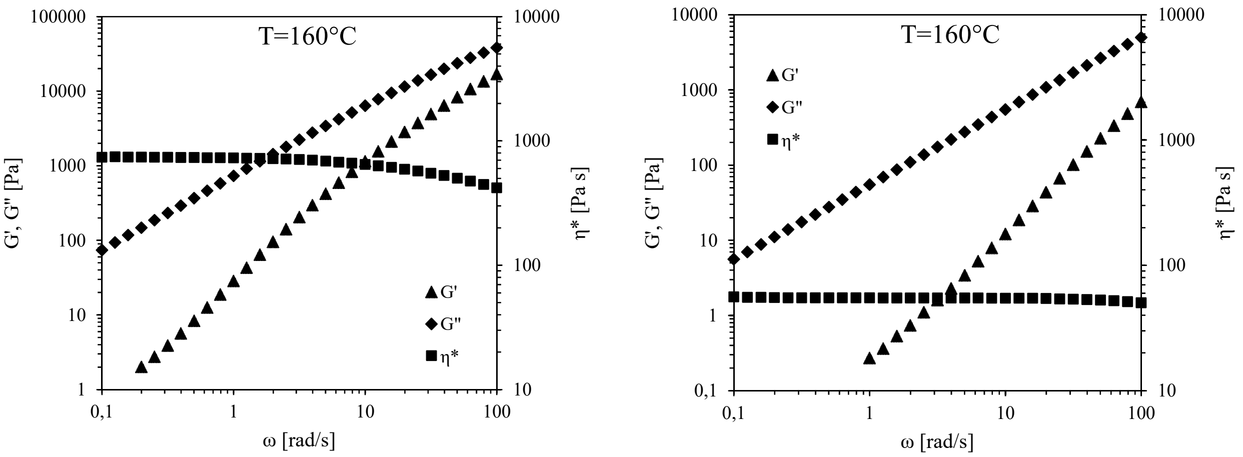


Figure S1. Oscillatory shear measurements in the frequency domain carried out at 160 °C on: (a) PBS, (b) PBAT.

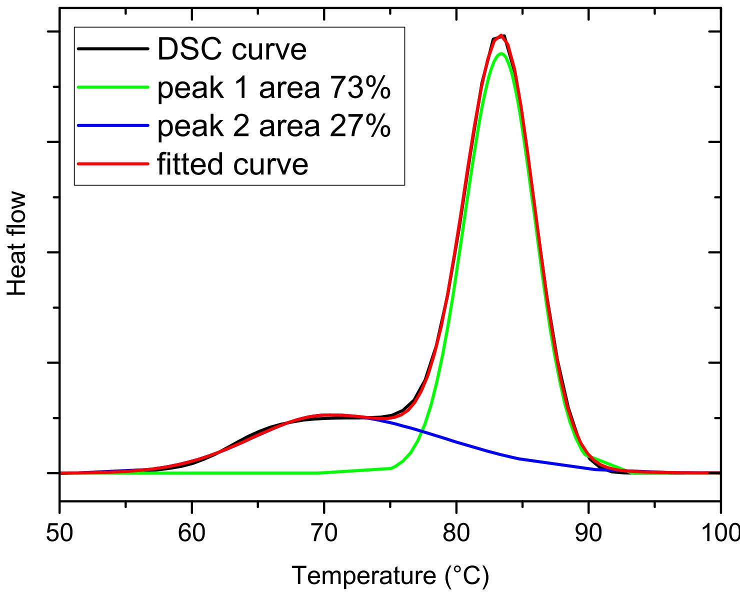


**Figure S2.** DSC crystallization peak deconvolution of PBS50-PBAT50 blend highlighting the PBS (green curve) and PBAT (blue curve) cooling crystallization peaks.

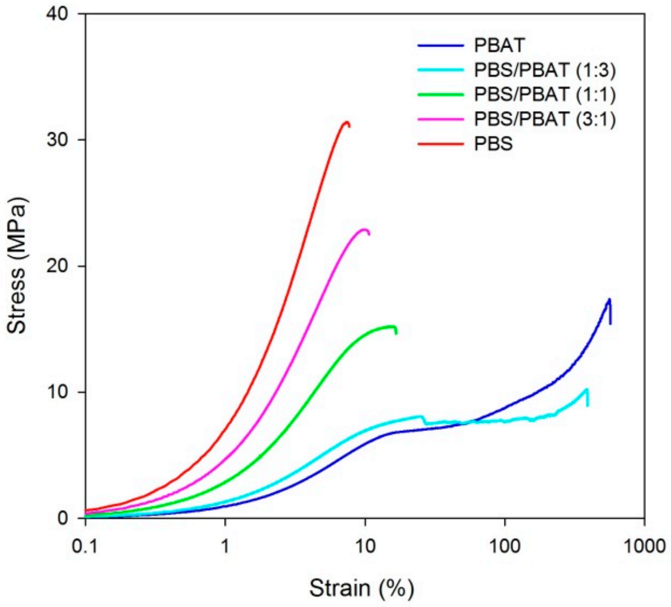


**Figure S3.** Illustrative stress-strain curves of films based on neat PBS, PBAT, and their blends.
